# SGLT2 Inhibitors and Hemoglobin Improvement in Myelodysplastic Syndromes and Myeloproliferative Neoplasms: Mechanistic Rationale and Emerging Clinical Evidence

**DOI:** 10.1155/anem/4918154

**Published:** 2026-11-08

**Authors:** Shehab F. Mohamed, Mohammed Abdulgayoom, Abdulrahman F. Al-Mashdali, Marrita Rabadi, Elabbass A. Abdelmahmuod, Mohamed Yassin

**Affiliations:** ^1^ Department of Hematology National Center for Cancer Care and Research (NCCCR) Hamad Medical Corporation Doha Qatar hamad.qa; ^2^ College of Medicine Qatar University Doha Qatar qu.edu.qa; ^3^ Qatar Metabolic Institute Hamad Medical Corporation Doha Qatar hamad.qa

**Keywords:** anemia, erythropoiesis, myelodysplastic syndromes, myelofibrosis, myeloproliferative neoplasms, SGLT2 inhibitors

## Abstract

Anemia represents one of the most clinically significant and therapeutically challenging complications in myelodysplastic syndromes (MDS) and myeloproliferative neoplasms (MPN), particularly in myelofibrosis. It is associated with increased symptom burden, transfusion dependence, and reduced overall survival. Sodium‐glucose cotransporter‐2 (SGLT2) inhibitors have consistently demonstrated increases in hemoglobin levels across multiple large randomized clinical trials in cardiovascular, renal, and metabolic diseases, raising interest in their potential role as modulators of erythropoiesis. This narrative review aimed to provide a comprehensive and mechanistically grounded overview of the role of SGLT2 inhibitors in improving hemoglobin levels in patients with MDS and MPN. A literature review was conducted using PubMed, Embase, and Google Scholar, focusing on the database from inception to June 2026. Studies evaluating SGLT2 inhibitors and hematologic outcomes in MDS, MDS/MPN overlap syndromes, myelofibrosis, or other MPNs were included. Both clinical and mechanistic studies were included. Current evidence in MDS and MPN remains limited to small observational cohorts, retrospective studies, and case reports. Across the available clinical studies in patients with MDS and MPN, SGLT2 inhibitors were associated with increases in hemoglobin, generally ranging from 1.0 to 1.6 g/dL. Specifically, a median peak hemoglobin increase of approximately 1.2 g/dL was reported among non‐transfusion‐dependent patients with MDS/MDS‐MPN overlap receiving SGLT2 inhibitors, while patients with myelofibrosis receiving predominantly empagliflozin or dapagliflozin had a median increase of 1.0 g/dL. In MDS and myelofibrosis, these findings may reflect improvement in ineffective erythropoiesis, whereas in non‐anemic MPN subtypes, the observed changes may instead represent erythrocytosis. Preliminary observational findings suggest a potential erythropoietic effect of SGLT2 inhibitors; however, whether this translates into a therapeutic benefit in MDS or MPN remains uncertain and requires prospective and mechanistic validation.

## 1. Introduction

Myelodysplastic syndromes (MDS) and myeloproliferative neoplasms (MPN) represent distinct yet biologically related groups of clonal hematopoietic stem cell disorders characterized by dysregulated hematopoiesis, variable cytopenias or cytoses, and a risk of progression to acute myeloid leukemia [1–3]. While these entities differ in their clinical manifestations and underlying biology, anemia is a common and clinically significant feature across both disease groups, particularly in MDS and in advanced forms of MPN such as myelofibrosis [1–3].

MDS are characterized by ineffective hematopoiesis resulting from clonal stem cell dysfunction, leading to peripheral blood cytopenias despite a typically hypercellular bone marrow [2, 3]. The pathogenesis involves a multistep process driven by somatic mutations affecting RNA splicing, epigenetic regulation, and transcriptional control, as well as alterations in the bone marrow microenvironment [3, 4]. These abnormalities lead to impaired erythroid differentiation and increased apoptosis of erythroid precursors, ultimately resulting in chronic anemia, the most frequent cytopenia in MDS [2, 3].

In contrast, MPNs are characterized by clonal proliferation of one or more myeloid lineages. Classical subtypes include polycythemia vera, essential thrombocythemia, and myelofibrosis, each with distinct clinical phenotypes [1, 5]. While polycythemia vera and essential thrombocythemia are typically associated with erythrocytosis and thrombocytosis, respectively, myelofibrosis is characterized by progressive bone marrow fibrosis, extramedullary hematopoiesis, and a high prevalence of anemia [1]. In myelofibrosis, anemia is multifactorial and reflects a combination of ineffective erythropoiesis, inflammatory cytokine‐mediated suppression, iron dysregulation, and treatment‐related effects [1, 6]. Importantly, anemia in this setting is associated with worse prognosis, increased symptom burden, and transfusion dependence, underscoring its clinical significance [1].

Despite advances in the understanding of disease biology, management of anemia in both MDS and MPN remains suboptimal. Current therapeutic strategies, including erythropoiesis‐stimulating agents (ESAs), transfusion support, and newer agents such as luspatercept, provide benefit in selected patients but are often limited by modest response rates, lack of durability, and restricted applicability based on disease characteristics [7, 8]. In myelofibrosis, treatment options are further complicated by the use of JAK inhibitors, which, while effective for symptom control and splenomegaly, may exacerbate anemia, particularly during early treatment phases [1, 9, 10]. An important exception is momelotinib, a JAK1/JAK2 and activin A receptor type 1 (ACVR1) inhibitor that suppresses hepcidin and improves iron availability and has shown clinically significant improvements in hemoglobin levels and transfusion independence in patients with myelofibrosis‐associated anemia [11]. Despite these advances, a substantial proportion of patients remain anemic or transfusion‐dependent, highlighting the need for new treatment strategies that address the underlying causes of anemia.

Sodium‐glucose cotransporter‐2 (SGLT2) inhibitors—including empagliflozin, dapagliflozin, and canagliflozin—have emerged as an important class of agents in the management of diabetes, heart failure, and chronic kidney disease [12–14]. Beyond their metabolic and cardiovascular benefits, these agents have consistently been associated with increases in hemoglobin and hematocrit across multiple large randomized clinical trials [12–14]. Initially attributed to hemoconcentration resulting from osmotic diuresis, subsequent mechanistic studies have demonstrated that these effects are largely mediated by stimulation of erythropoiesis, including increased erythropoietin production, improved renal oxygenation, and modulation of iron metabolism [12–16].

In this narrative review, we aim to critically review the available literature examining the association between SGLT2 inhibitor use and hemoglobin changes in MDS and MPN. This review discusses the pathophysiology of anemia in these disorders, the mechanistic rationale for a potential erythropoietic effect of SGLT2 inhibition, and the limitations of the currently available clinical evidence.

## 2. Methods

This study was conducted as a narrative review. A literature search was performed using PubMed, Embase, and Google Scholar from database inception through June 2026. Search terms included “SGLT2 inhibitors,” “hemoglobin,” “anemia,” “erythropoiesis,” “myelodysplastic syndromes,” and “myeloproliferative neoplasms,” along with specific drug names.

Relevant publications were identified based on clinical and scientific relevance and included randomized trials, observational studies, case series, and case reports reporting hematologic outcomes. Given the limited availability of direct evidence in MDS and MPN, mechanistic and translational studies were also considered to support biological plausibility.

Studies were selected and interpreted qualitatively, with emphasis on study design, patient population, intervention characteristics, and reported hemoglobin outcomes. Findings were synthesized narratively to provide a comprehensive overview of the current evidence.

### 2.1. Clinical Evidence

No randomized controlled trials evaluating SGLT2 inhibitors specifically for anemia in MDS, MDS/MPN, or MPN were identified. The available evidence is therefore limited to small retrospective cohorts, case series, and individual case reports, with substantial heterogeneity in disease subtype, baseline anemia status, prior therapies, SGLT2 inhibitor exposure, and outcome reporting (Table 1) [17–22]. Collectively, the published literature includes approximately 58 patients exposed to SGLT2 inhibitors across MDS, MDS/MPN overlap syndromes, myelofibrosis, essential thrombocythemia, and polycythemia vera. Despite these limitations, the available literature provides a consistent signal that SGLT2 inhibitors are associated with increases in hemoglobin and hematocrit, although the clinical meaning of this effect differs substantially between anemic and non‐anemic myeloid neoplasms.

**TABLE 1:** Clinical evidence of SGLT2 inhibitors in MDS and MPN.

Study (ref)	Country	Disease	*N*	Study type	Population	SGLT2 inhibitor	Baseline Hb (g/dL)	Hb change (g/dL)	Hct change	Response rate (%)	Follow‐up duration	Key findings	Clinical interpretation
Das et al. (2018) [22]	USA	Polycythemia vera	1	Case report	Non‐anemic PV	Canagliflozin	14.5	+2.4	∼+7%	N/A	Case report	Rapid rise in Hb/Hct leading to PV diagnosis	Safety signal (erythrocytosis)
Yamasaki et al. (2023) [17]	Japan	MDS (TD)	3	Case series	Severe anemia	Dapagliflozin + daprodustat	6–8	+2–3.5	NR	100%	NR	Transfusion independence achieved	Combination effect
Patır et al. (2024) [21]	Turkey	Mixed MPN	16	Case series	Mostly non‐anemic	Mixed	13.5	+0.7	+1.7%	NR	NR	Some required phlebotomy	Monitoring needed
Aperna et al. (2025) [18]	USA	Myelofibrosis	16	Cohort	Anemic MF	Mixed	11.0	+1.0 (0–3.0)	+4%	88% (14/16)	NR	Durable Hb responses	Strongest MF evidence
Alsugair et al. (2025) [19]	Multicenter	MDS/MDS‐MPN	11	Cohort	Anemic	Empagliflozin/dapagliflozin	9.6	+1.2 peak	+3%	67%	NR	≥ 1 g/dL Hb increase	Best MDS evidence
Gangat et al. (2023) [20]	USA	ET	11	Case series	Mostly nonanemic	Mixed	12.8	+1.5	+5%	NR	NR	Hb/Hct increase	Erythropoietic effect

*Note:* Hb, hemoglobin; Hct, hematocrit; MF, myelofibrosis.

Abbreviations: ET, essential thrombocythemia; NR, not reported; TD, transfusion‐dependent.

In patients with MDS and MDS/MPN overlap syndromes, the most directly relevant evidence comes from a retrospective study that evaluated 11 anemic patients receiving SGLT2 inhibitors [19]. Among the nine non‐transfusion‐dependent patients, the median increase in hemoglobin from baseline to peak was 1.2 g/dL, and six patients (67%) achieved a hemoglobin increase of ≥ 1 g/dL. The corresponding median increase in hematocrit from baseline to peak was 3.7%, further supporting a measurable erythropoietic effect. Importantly, interpretation of these findings should account for concomitant therapy and baseline transfusion status. Of the 11 evaluable patients, four were receiving stable MDS‐ or anemia‐directed therapy for > 3 months before SGLT2 inhibitor initiation (darbepoetin, *n* = 2; luspatercept, *n* = 1; azacitidine, *n* = 1), six were treatment‐naïve, and one had previously received luspatercept and lenalidomide, which had been discontinued before SGLT2 inhibitor initiation. Two patients were transfusion‐dependent at baseline, and neither responded. Serum erythropoietin levels were not systematically assessed and were available in only five patients, all of whom were transfusion‐independent (median, 22.3 mIU/mL; range, 14.6–62.2); three were responders and two were nonresponders [19]. Given the limited number of informative cases, no association between pre‐treatment erythropoietin levels and hemoglobin response could be established. These factors, particularly concomitant anemia‐directed therapy, should be considered when attributing the observed hemoglobin improvements specifically to SGLT2 inhibition.

Additional evidence in MDS is provided by a study reporting three patients with transfusion‐dependent lower risk MDS treated with dapagliflozin in combination with daprodustat, a hypoxia‐inducible factor prolyl‐hydroxylase inhibitor [17]. All patients reportedly achieved improvement in anemia and transfusion independence, with hemoglobin increases in the range of approximately 2–3.5 g/dL. However, this report requires cautious interpretation because the intervention combined an SGLT2 inhibitor with daprodustat, an agent with a direct erythropoietic mechanism. Therefore, the observed benefit cannot be attributed solely to dapagliflozin. Nevertheless, the magnitude of response supports the concept that SGLT2 inhibition may complement other erythropoiesis‐enhancing strategies in selected patients with residual marrow capacity [17].

In myelofibrosis, the strongest disease‐specific signal comes from a study that reported hemoglobin changes in 16 patients receiving SGLT2 inhibitors, comprising 12 patients with primary myelofibrosis and four with post‐essential thrombocythemia myelofibrosis [18]. In this cohort, median age was 74 years, and median baseline hemoglobin was 11 g/dL (range, 8–15); six patients (38%) had hemoglobin < 10 g/dL, and one had transfusion‐dependent anemia. Splenomegaly was present in 10 patients (63%), although quantitative spleen measurements were not reported. Seven patients (44%) were receiving cytoreductive therapy, including hydroxyurea (*n* = 2), ruxolitinib (*n* = 4), and momelotinib (*n* = 1), while two patients received darbepoetin alfa. The study showed that 14 of 16 (88%) experienced an increase in hemoglobin after SGLT2 inhibitor initiation; nine patients (54%) had an increase of ≥ 1 g/dL, and six (38%) had an increase of ≥ 1.5 g/dL. The median hemoglobin increase was 1.0 g/dL (range, 0–3.0 g/dL), while the median hematocrit increase was 4% (range, 0–11%). Among the six patients with baseline hemoglobin < 10 g/dL, five (83%) achieved an increase of ≥ 1 g/dL [18]. Constitutional symptom burden and formal prognostic‐risk categories were not reported in the published cohort. These findings are clinically meaningful because anemia is a central feature of myelofibrosis and is associated with adverse prognosis, symptom burden, and transfusion dependence. The observation of sustained hemoglobin improvement in a subset of patients suggests that SGLT2 inhibitors may enhance residual erythropoiesis in myelofibrosis, although the uncontrolled design precludes firm conclusions regarding causality.

The interpretation of hemoglobin increases differs in non‐anemic MPN subtypes. A study evaluated 11 patients with essential thrombocythemia receiving SGLT2 inhibitors and observed increases in hemoglobin and hematocrit, with a median baseline hemoglobin of approximately 12.8 g/dL and a post‐treatment increase of approximately 1.5 g/dL [20]. Because these patients were predominantly non‐anemic at baseline, this finding should not be interpreted as evidence of anemia correction. Rather, it supports a broader erythropoietic effect of SGLT2 inhibitors and raises the possibility of clinically relevant erythrocytosis in susceptible MPN populations [20].

Similarly, a case series of 16 patients with MPN receiving SGLT2 inhibitors showed that most patients were not anemic at baseline [21]. The median hemoglobin increase was modest, approximately 0.7 g/dL, with a hematocrit increase of approximately 1.7%. Although no major uniform anemia benefit was demonstrated, some patients required therapeutic intervention such as phlebotomy, emphasizing that hemoglobin and hematocrit increases may be clinically relevant even when they do not represent a therapeutic anemia response. This distinction is important when evaluating SGLT2 inhibitors across MPN subtypes, because an erythropoietic effect may be desirable in myelofibrosis‐associated anemia but potentially problematic in polycythemia vera or essential thrombocythemia.

A safety‐related case report further supports this cautionary interpretation. In that report, canagliflozin was associated with a marked rise in hemoglobin from approximately 14.5 g/dL to 16.9 g/dL and an increase in hematocrit from approximately 48% to 55%, ultimately leading to the diagnosis of underlying polycythemia vera [22]. This case suggests that SGLT2 inhibitors may unmask or exacerbate latent clonal erythrocytosis in predisposed individuals. Although a single case cannot establish causality, it provides an important safety signal and supports careful monitoring of hemoglobin and hematocrit in patients with MPN or unexplained erythrocytosis receiving SGLT2 inhibitors [22].

Collectively, the evidence suggests that SGLT2 inhibitors exert a reproducible erythropoietic effect across diverse hematologic contexts. In anemic diseases such as MDS, MDS/MPN overlap syndromes, and myelofibrosis, this effect may translate into clinically meaningful hemoglobin improvement. In contrast, in non‐anemic MPN populations such as essential thrombocythemia and polycythemia vera, the same biological effect may manifest as erythrocytosis rather than anemia correction. Therefore, the available evidence supports a disease‐context–specific interpretation: SGLT2 inhibitors may be potentially beneficial in patients with baseline anemia and residual erythropoietic capacity but require caution in patients with preserved or elevated hemoglobin at baseline [17–22]. Overall, the current evidence base remains limited by small sample sizes, retrospective study designs, lack of control groups, and heterogeneity in patient populations and concomitant therapies (Table 1). Consequently, the observed hemoglobin improvements should be interpreted as hypothesis‐generating rather than definitive evidence of efficacy, emphasizing the need for prospective clinical trials.

## 3. Discussion

### 3.1. Myelofibrosis as an Anemia‐Predominant MPN

Myelofibrosis represents a biologically and clinically distinct entity within the spectrum of MPN [23, 24]. Unlike polycythemia vera and essential thrombocythemia, which are characterized by erythrocytosis and thrombocytosis, respectively, myelofibrosis is predominantly an anemia‐driven disease characterized by progressive bone marrow fibrosis, extramedullary hematopoiesis, and ineffective erythropoiesis [1, 25].

Anemia is present in approximately one‐third to one‐half of patients at diagnosis [1, 6, 23, 25]. Importantly, it is one of the strongest adverse prognostic factors, incorporated into multiple prognostic scoring systems, and is associated with reduced survival, increased symptom burden, and higher transfusion dependence [1, 25].

### 3.2. Pathophysiology of Anemia in Myelofibrosis

The development of anemia in myelofibrosis is multifactorial and reflects a complex interplay between intrinsic clonal hematopoiesis and extrinsic microenvironmental factors. Progressive fibrosis of the bone marrow leads to disruption of the hematopoietic niche, impairing erythroid progenitor proliferation and differentiation [1, 25].

In parallel, a pro‐inflammatory cytokine milieu plays a central role. Elevated levels of interleukin‐6, tumor necrosis factor‐α, and other inflammatory mediators suppress erythropoiesis and contribute to anemia of chronic disease [25, 26]. These cytokines also stimulate hepatic hepcidin production, resulting in functional iron deficiency through sequestration of iron within macrophages and decreased intestinal iron absorption [26, 27].

Additional mechanisms include extramedullary hematopoiesis, particularly in the spleen, leading to sequestration and destruction of circulating erythrocytes, as well as shortened red blood cell survival. Furthermore, therapeutic interventions such as JAK inhibitors (e.g., ruxolitinib) may exacerbate anemia, particularly during early phases of treatment, likely due to suppression of erythropoietic signaling pathways [9]. Collectively, these mechanisms result in ineffective erythropoiesis and inadequate compensation, leading to clinically significant anemia.

### 3.3. Current Management of Anemia in Myelofibrosis

Management of anemia in myelofibrosis remains a major unmet clinical need. Current therapeutic approaches are largely supportive and include ESAs, androgens such as danazol, corticosteroids, and immunomodulatory agents including thalidomide and lenalidomide, although response rates are variable and frequently transient [1, 28].

ESAs are commonly used in patients with low endogenous erythropoietin levels and may provide temporary improvement in hemoglobin levels and transfusion requirements. However, responses are often modest and less effective in patients with advanced disease or transfusion dependence [3].

Danazol, a synthetic androgen, has demonstrated clinical activity in myelofibrosis‐associated anemia and may improve hemoglobin levels in selected patients [9, 28, 29]. Its mechanism is thought to involve stimulation of erythropoiesis and modulation of inflammatory cytokines. Nevertheless, long‐term use may be limited by hepatic toxicity and other androgen‐related adverse effects, requiring liver and prostate monitoring [29].

Immunomodulatory agents such as thalidomide and lenalidomide have also been evaluated in myelofibrosis‐associated anemia, particularly in combination with corticosteroids. While some patients experience hematologic improvement, these therapies are frequently associated with significant toxicity, including neuropathy, cytopenias, and poor tolerability in older adults [30].

More recently, luspatercept, an activin receptor ligand trap that enhances late‐stage erythroid maturation, has shown promising activity in selected patients with myelofibrosis‐associated anemia, particularly those with features of ineffective erythropoiesis or transfusion dependence [31]. Early clinical studies demonstrated reductions in transfusion burden and improvement in hemoglobin levels, especially when combined with JAK inhibitor therapy [8, 32].

JAK inhibitors, including ruxolitinib and fedratinib, remain the cornerstone of therapy for splenomegaly and constitutional symptom control in myelofibrosis [9, 33]. However, their use is commonly associated with worsening anemia, particularly during treatment initiation, likely due to on‐target suppression of erythropoietin signaling pathways [9].

In contrast, momelotinib, a JAK1/JAK2 and activin A receptor type 1 (ACVR1) inhibitor, has emerged as a particularly important therapeutic option for patients with myelofibrosis‐associated anemia. Beyond spleen and symptom control, momelotinib has demonstrated clinically meaningful improvements in transfusion independence and hemoglobin levels [34]. Its anemia benefit is believed to result from suppression of hepcidin production through ACVR1 inhibition, thereby improving iron availability and erythropoiesis [15]. These properties distinguish momelotinib from other JAK inhibitors and make it especially attractive for anemic patients with myelofibrosis.

Imetelstat, a first‐in‐class telomerase inhibitor, has also demonstrated promising activity in patients with relapsed or refractory myelofibrosis. In addition to potential disease‐modifying effects and improvement in overall survival observed in clinical studies, imetelstat may contribute indirectly to anemia improvement in selected patients through reduction of malignant clonal burden and bone marrow fibrosis [16]. However, treatment‐related cytopenias remain common, and their precise role in anemia management continues to evolve.

Despite these advances, anemia in myelofibrosis remains difficult to manage, and many patients require transfusion support. The limitations of currently available therapies highlight the ongoing need for novel treatment approaches capable of improving erythropoiesis while maintaining disease control.

### 3.4. Pathophysiology of Anemia in MDS

Anemia in MDS is primarily driven by ineffective erythropoiesis resulting from clonal hematopoietic stem cell dysfunction [2, 3]. In contrast to myelofibrosis, the bone marrow in MDS is often hypercellular, yet fails to produce adequate mature erythrocytes due to increased apoptosis of erythroid precursors and impaired differentiation [35].

This process is driven by a spectrum of genetic and epigenetic abnormalities, including mutations affecting RNA splicing (e.g., SF3B1), DNA methylation (e.g., TET2, DNMT3A), and transcriptional regulation, which disrupt normal erythroid maturation pathways [3, 36].

Ineffective hematopoiesis and MDS pathophysiology are also significantly influenced by dysregulated innate immune signaling and chronic inflammation. Patients with MDS have been shown to have elevated levels of pro‐inflammatory cytokines, including TNF‐α, IL‐6, IL‐1β, and IFN‐γ, which can promote apoptosis and adversely affect hematopoietic stem and progenitor cell survival [37, 38]. At the molecular level, aberrant activation of innate immune pathways, particularly TLR/MyD88/NF‐κB signaling and the S100A8/S100A9–NLRP3 inflammasome axis, contributes to inflammatory signaling, pyroptotic cell death, and ineffective hematopoiesis in MDS [39]. Additionally, inflammation can worsen anemia through IL‐6–mediated induction of hepcidin, which limits iron availability for erythropoiesis [40].

Additionally, alterations in the bone marrow microenvironment and increased inflammatory signaling further suppress erythropoiesis [36]. Dysregulation of iron metabolism, including elevated hepcidin levels, contributes to functional iron deficiency, while chronic transfusion therapy may lead to iron overload, further complicating disease management [26, 27].

### 3.5. Current Management of Anemia in MDS

Anemia treatment in lower risk MDS is becoming more personalized based on cytogenetic and molecular characteristics, serum erythropoietin level, transfusion dependency, and previous therapy. ESAs remain an established first‐line approach for symptomatic, transfusion‐independent or lower transfusion‐burden patients, particularly individuals with lower endogenous erythropoietin levels [41]. However, in ESA‐naïve, transfusion‐dependent lower risk MDS, the phase III COMMANDS trial showed superior erythroid responses and red blood cell transfusion independence with luspatercept as opposed to epoetin alfa, establishing luspatercept as an important first‐line option in this setting, including in patients without ring sideroblasts or SF3B1 mutations [42].

Disease‐specific characteristics should also be considered in the treatment selection process. For patients with transfusion‐dependent lower risk MDS with del(5q), lenalidomide remains the preferred disease‐directed treatment because of its high rates of erythroid response and transfusion independence [43]. For patients with transfusion‐dependent, non‐del(5q) lower risk MDS who are refractory to, have relapsed after, or are ineligible for ESAs, imetelstat represents an additional therapeutic option [44]. In the phase III IMerge trial, 8‐week transfusion independence was achieved in 40% of patients receiving imetelstat compared with 15% receiving placebo. Following luspatercept, treatment sequencing is still less well‐established and should be customized based on patient considerations, disease manifestations, transfusion burden, and previous therapy.

Iron chelation and red blood cell transfusions remain crucial parts of supportive care, while hypomethylating agents and other disease‐modifying approaches may be considered depending on the patient’s risk profile and clinical condition [45].

### 3.6. Mechanistic Rationale for SGLT2 Inhibitors in MDS and Myelofibrosis

SGLT2 inhibitors, including empagliflozin, dapagliflozin, and canagliflozin, have consistently been associated with increases in hemoglobin and hematocrit across multiple large randomized trials in diabetes, heart failure, and chronic kidney disease [12–14, 46].

Importantly, these randomized cardiovascular and renal outcome trials have consistently shown the erythropoietic effects of SGLT2 inhibitors. Empagliflozin was associated with long‐term increases in hemoglobin and hematocrit in the EMPA‐REG OUTCOME study [14]; these results were subsequently found to be among the most significant mediators of the observed cardiovascular benefit. The DAPA‐HF [13] and EMPEROR heart failure trials [47], as well as the CREDENCE trial assessing canagliflozin in chronic renal disease [12], revealed similar hematologic outcomes. Interestingly, similar increases were seen in a variety of patient demographics and occurred regardless of the patient’s initial anemia status, indicating a class impact rather than a phenomenon specific to the disease [12–16, 48].

Initially attributed to hemoconcentration resulting from osmotic diuresis, subsequent mechanistic studies have demonstrated that these effects reflect true stimulation of erythropoiesis. SGLT2 inhibition leads to increased erythropoietin production, likely mediated by improved renal oxygenation and restoration of tubulointerstitial function [15, 48].

In addition, SGLT2 inhibitors may modulate iron metabolism through suppression of the hepcidin axis, potentially improving iron availability for erythropoiesis [26, 27]. In a randomized trial, erythroferrone, a physiological suppressor of hepatic hepcidin expression, increased by approximately 22% after 12 weeks of dapagliflozin administration, whereas circulating hepcidin concentrations decreased by roughly 24% from baseline [16]. These findings support a true biological suppressive effect on hepcidin production rather than a reduction in circulating hepcidin attributable solely to changes in plasma volume.

In myelofibrosis, ACVR1‐mediated hepcidin upregulation has emerged as an important mechanism of iron‐restricted anemia. Chronically elevated hepcidin limits intestinal iron absorption and macrophage iron release, reducing iron availability for erythropoiesis [49]. This pathway is clinically relevant, as ACVR1 inhibition by agents such as momelotinib and pacritinib has been associated with reduced hepcidin signaling and improvement in anemia outcomes [30, 32].

SGLT2 inhibitors may fit into this biological framework through a complementary mechanism. Although they do not directly inhibit ACVR1, they may enhance erythropoietin‐driven erythropoiesis while partially reversing hepcidin‐mediated iron restriction. This provides a plausible explanation for the observed hemoglobin improvement in patients with myelofibrosis receiving SGLT2 inhibitors and supports further investigation of this pathway.

Recent evidence has further expanded the potential role of SGLT2 inhibitors in disorders characterized by chronic inflammation and ineffective erythropoiesis. A comprehensive review highlighted that SGLT2 inhibitors may favorably modulate pathways implicated in anemia of chronic disease, including pro‐inflammatory cytokine signaling, particularly IL‐6 and TNF‐α, hepcidin‐mediated iron restriction, and erythropoietic regulation [46]. Although direct evidence remains limited, the convergence of these pathways suggests that SGLT2 inhibition may exert effects extending beyond simple hemoconcentration and may contribute to meaningful improvements in erythropoietic efficiency in selected patients [46].

Despite important differences in disease biology, the available mechanistic and clinical evidence suggests that SGLT2 inhibitors may improve hemoglobin levels in both myelofibrosis and selected patients with MDS. The potential benefit appears most biologically plausible in myelofibrosis, where reversible mechanisms such as inflammation and iron restriction contribute substantially to anemia, whereas in MDS, any benefit is likely dependent on the presence of residual erythropoietic capacity (Figures 1 and 2).

**FIGURE 1:**
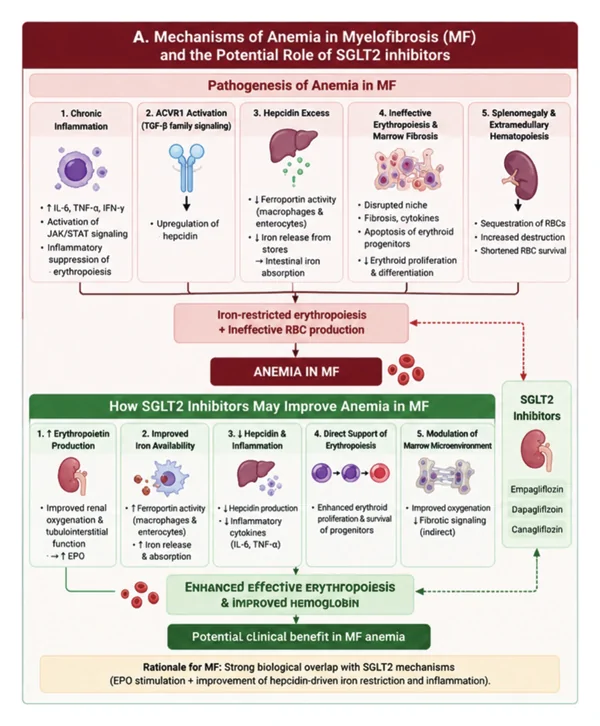
Mechanisms of anemia in myelofibrosis and the potential role of SGLT2 inhibitors.

**FIGURE 2:**
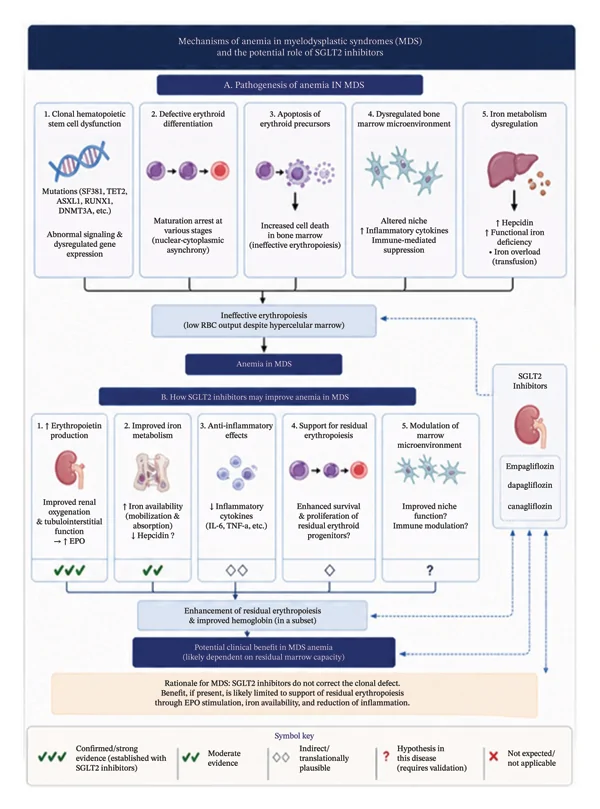
Proposed mechanisms of anemia in myelodysplastic syndromes and potential role of SGLT2 inhibitors. Anemia in MDS results from clonal hematopoietic stem‐cell dysfunction, ineffective erythropoiesis, impaired erythroid maturation, and increased apoptosis of erythroid precursors, with important contributions from inflammatory signaling and dysregulated iron metabolism. Through enhanced erythropoietin signaling and control of hepcidin‐mediated iron restriction, SGLT2 inhibitors may improve erythropoiesis; nevertheless, their potential impact is likely dependent on preserved residual erythropoietic capacity. Abbreviations: MDS, myelodysplastic syndromes; SGLT2, sodium‐glucose cotransporter‐2; EPO, erythropoietin.

### 3.7. Clinical Evidence Interpretation

Importantly, no randomized controlled trials have evaluated SGLT2 inhibitors in MDS or MPN, and the current evidence is entirely derived from small observational cohorts, case series, and case reports. This significantly limits the strength of inference and underscores the exploratory nature of the available data.

Despite these limitations, a consistent signal emerges across studies. In MDS and MDS/MPN overlap syndromes, SGLT2 inhibitor use is associated with modest but clinically meaningful increases in hemoglobin, typically in the range of 1.0–1.2 g/dL, with some patients achieving sustained responses and, in select cases, transfusion independence [17, 19]. In myelofibrosis, hemoglobin improvement was reported in 14 of 16 patients (88%) receiving SGLT2 inhibitors, with a median hemoglobin increase of 1.0 g/dL [18]. However, the clinical interpretation of these findings differs across disease subtypes. In MDS and myelofibrosis, hemoglobin increases may reflect a therapeutic effect in the context of baseline anemia. In contrast, in other MPN subtypes such as essential thrombocythemia and polycythemia vera, reported increases in hemoglobin and hematocrit are more consistent with an erythropoietic or erythrocytosis effect rather than correction of anemia [20, 21]. Chronic inflammation, hepcidin‐mediated iron restriction, and inflammatory suppression of erythropoiesis contribute to anemia in myelofibrosis [50, 51]. SGLT2 inhibitors have been shown to modulate erythropoietin production, iron metabolism, hepcidin, and inflammatory biomarkers; however, whether these mechanisms mediate the observed hemoglobin increases in myelofibrosis remains unproven. Importantly, inflammatory and innate immune dysregulation also contribute to ineffective hematopoiesis in MDS, alongside intrinsic clonal, genetic, and epigenetic abnormalities [37–39]. Therefore, although these pathways provide biological plausibility for investigating SGLT2 inhibition in both MDS and myelofibrosis, their contribution to the observed clinical responses remains to be established.

Variability in response across studies is also evident. While many cohorts demonstrate meaningful hemoglobin improvement, others report modest or minimal changes, and in some cases, increases in hematocrit requiring clinical intervention. This heterogeneity likely reflects differences in baseline anemia severity, disease biology, residual marrow function, and concomitant therapies. Notably, a study employed combination therapy with dapagliflozin and daprodustat, limiting the ability to attribute the observed hematologic improvements solely to SGLT2 inhibition and highlighting the need for cautious interpretation of combination approaches [17].

Safety considerations are particularly important. Published meta‐analyses have reported the adverse effects of this drug class, which include genital mycotic infections, urinary tract infections, volume depletion and hypotension, and, rarely, euglycemic diabetic ketoacidosis [52, 53]. From a hematologic perspective, the increase in hemoglobin and hematocrit associated with SGLT2 inhibition also warrants attention. A reported case of marked erythrocytosis leading to the diagnosis of polycythemia vera suggests that SGLT2 inhibitors may unmask or exacerbate underlying clonal erythrocytosis in susceptible individuals [22]. In patients with MPN, excessive hematocrit elevation may be particularly concerning in polycythemia vera, in which maintaining hematocrit below 45% significantly reduces cardiovascular death and major thrombosis [54].

### 3.8. Clinical Implications

The emerging evidence suggests that SGLT2 inhibitors may have a potential role in the management of anemia in selected patients with MDS and MPN, particularly those with baseline anemia and preserved marrow function. Across available studies, the most consistent signal of benefit has been observed in lower risk MDS and myelofibrosis, where modest but clinically meaningful increases in hemoglobin have been reported, with some patients achieving reduced transfusion requirements or transfusion independence.

Selection of patients is crucial. Patients with preserved residual erythropoietic capacity, transfusion dependence, or symptomatic anemia may be more likely to benefit, although predictors of response remain undefined. On the other hand, increases in hemoglobin and hematocrit may indicate erythrocytosis rather than anemia correction; therefore, use in non‐anemic patients—especially those with essential thrombocythemia or polycythemia vera—should be done cautiously. Baseline evaluation should include hemoglobin level, transfusion burden, disease subtype and risk category, iron status, renal function, thrombotic risk, and concomitant therapies that may influence hematologic response. SGLT2 inhibitors should be prescribed according to standard dosing recommendations with appropriate consideration of renal function and tolerability.

Close monitoring is also essential, particularly during the early phases of treatment. Hemoglobin and hematocrit should be assessed regularly, alongside evaluation for symptoms of hyperviscosity or volume depletion. In patients demonstrating excessive increases in hematocrit, dose adjustment, temporary discontinuation, or standard interventions such as phlebotomy may be required (Figure 3).

**FIGURE 3:**
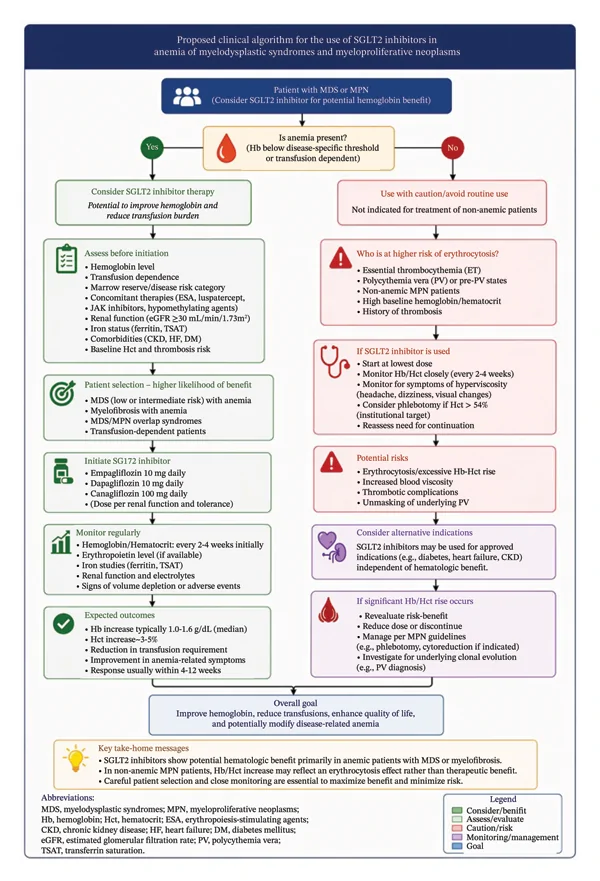
Hypothesis‐generating clinical algorithm for considering SGLT2 inhibitors in patients with MDS and MPN. The algorithm outlines key clinical considerations, including baseline anemia, transfusion burden, iron status, renal function, and thrombotic risk, with monitoring of hemoglobin and hematocrit during therapy. This framework is hypothesis‐generating and does not represent an established treatment guideline. Abbreviations: MDS, myelodysplastic syndromes; MPN, myeloproliferative neoplasms; SGLT2, sodium‐glucose cotransporter‐2; Hb, hemoglobin; Hct, hematocrit.

Importantly, SGLT2 inhibitors should not currently be considered standard therapy for anemia in MDS or MPN but rather as a potential adjunct in carefully selected patients. In individuals with coexisting indications such as diabetes, chronic kidney disease, or heart failure, these agents may offer dual clinical benefit, supporting their consideration within a broader therapeutic strategy.

### 3.9. Limitations

This review has several limitations. The available evidence consists predominantly of retrospective cohorts, case series, and case reports, with substantial heterogeneity in disease subtype, baseline anemia severity, concomitant therapies, and outcome reporting. Hence, a systematic review was not feasible. Publication and reporting bias cannot be excluded, as favorable hematologic responses may be more likely to be reported. Furthermore, the absence of randomized controlled trials precludes definitive conclusions regarding efficacy, safety, or causality.

### 3.10. Future Directions

Future research should prioritize phase II clinical trials to determine whether SGLT2 inhibitors can produce clinically meaningful and durable hemoglobin improvement in patients with MDS, MDS/MPN overlap syndromes, and myelofibrosis. Particular emphasis should be placed on identifying predictors of response, including disease subtype, transfusion burden, endogenous erythropoietin levels, iron parameters, inflammatory markers, hepcidin levels, mutational profile, and residual erythropoietic capacity.

Combination strategies with established anemia‐directed therapies, including ESAs, luspatercept, hypoxia‐inducible factor prolyl‐hydroxylase inhibitors, and JAK inhibitors, warrant further investigation. Future studies should incorporate clinically meaningful endpoints beyond hemoglobin change alone, including transfusion independence, transfusion burden, quality of life, symptom improvement, duration of response, and survival outcomes. Standardized definitions of hematologic response will be essential to facilitate comparison across studies.

A clearer mechanistic understanding is also needed before the observed hemoglobin increases can be interpreted as therapeutic efficacy. Future studies should determine whether these effects are mediated by erythropoietin stimulation, hepcidin suppression, and improved iron availability, or modulation of inflammatory signaling, and correlate these mechanisms with hematologic responses in MDS and MPN.

Given the potential for SGLT2 inhibitors to increase hemoglobin and hematocrit, careful evaluation of thrombotic risk, hyperviscosity symptoms, and erythrocytosis is required, particularly in patients with essential thrombocythemia or polycythemia vera. Mechanistic studies should further define the effects of SGLT2 inhibition on erythropoietin production, iron metabolism, hepcidin regulation, inflammation, marrow fibrosis, and clonal hematopoiesis in myeloid neoplasms.

## 4. Conclusion

SGLT2 inhibitors have emerged as a biologically plausible approach for modulating erythropoiesis in MDS, MDS/MPN overlap syndromes, and myelofibrosis. However, the clinical evidence to date remains preliminary, stemming primarily from small observational cohorts, case series, and case reports without randomized prospective validation. Although hemoglobin increases have been reported in some anemic patients, these findings are hypothesis‐generating and cannot currently prove therapeutic efficacy or causality. These effects may also be context‐dependent across clonal myeloid disorders, with potential benefit in anemic states but uncertain or potentially undesirable effects in non‐anemic MPN phenotypes. Mechanistically, impacts on iron availability, hepcidin regulation, erythropoietin production, and inflammatory signaling justify further investigation, especially considering the role of innate immune dysregulation and chronic inflammation in ineffective hematopoiesis in MDS. Prospective controlled studies incorporating mechanistic biomarkers are therefore needed to determine whether SGLT2 inhibition directly improves erythropoiesis, identify patients most likely to benefit, and establish long‐term efficacy and safety before these agents can be considered for anemia‐directed therapy in clonal myeloid disorders.

## Author Contributions

Shehab F. Mohamed and Mohammed Abdulgayoom conceived and designed the review. Shehab F. Mohamed and Marrita Rabadi conducted the literature search and drafted the manuscript. Abdulrahman F. Al‐Mashdali, Elabbass A. Abdelmahmuod, and Mohamed Yassin provided critical revision of the manuscript and supervised the paper. Mohammed Abdulgayoom had full access to all of the data in this study and takes complete responsibility for the integrity of the data and the accuracy of the data analysis.

## Funding

The publication charges for this article were covered by the Qatar National Library (QNL). No other specific funding was received for this work.

## Disclosure

All authors have read and approved the final version of the manuscript.

## Conflicts of Interest

The authors declare no conflicts of interest.

## Data Availability

This review is based on data extracted from previously published case reports, case series, cohort studies, and randomized controlled trials, all of which are cited in the manuscript and tables.
